# Emerging collaboration amid the COVID-19 within the context of traditional-state dualism governance in Bali

**DOI:** 10.4102/jamba.v16i1.1581

**Published:** 2024-04-29

**Authors:** Vanda Ningrum, Bambang S. Laksmono, Cahyo Pamungkas, Renny Nurhasana, Inayah Hidayati, Luh Kitty Katherina

**Affiliations:** 1 School of Strategic and Global Studies, Universitas Indonesia, Jakarta, Indonesia; 2 Research Center for Population, National Research and Innovation Agency, Jakarta, Indonesia; 3 Department of Social Welfare, Faculty of Social and Political Sciences, Universitas Indonesia, Depok, Indonesia; 4 Research Center for Area Studies, National Research and Innovation Agency, Jakarta, Indonesia; 5 Urban Studies Program, School of Strategic and Global Studies, Universitas Indonesia, Jakarta, Indonesia

**Keywords:** COVID-19, disaster management, dualism governance, Bali, Indonesia

## Abstract

Collaboration between government and communities in disaster management has been demonstrated to create disaster risk reduction initiatives. However, implementing such collaboration poses challenges within regions characterised by dualism in governance. This study aims to investigate the disaster management system for coronavirus disease 2019 (COVID-19) in the context of dualism governance, where the state holds administrative control. Traditional authority (*Adat*) simultaneously regulates various facets of human life in Bali, Indonesia. The study used a qualitative approach, and data were gathered through in-depth interviews with 10 informants, 3 rounds of focus group discussions, and participant observations. The findings reveal that the dualism governance in Bali is manifested through responses to vulnerabilities within the community, *Adat* versus state laws, and the communication systems established between state administrative and traditional authorities during the COVID-19 response.

**Contribution:**

This study highlights the significant role of traditional governance in enhancing community resilience amid the limitations of state capacity in handling the COVID-19 pandemic. Despite conflicting interests with the state, traditional government plays a crucial role in fostering collective community action to address vulnerabilities. The study underscores the importance of greater involvement of *Adat* actors in disaster management within the context of dualism in governance, spanning from mitigation planning to preparedness, response and recovery. This involvement has the potential to bolster community resilience.

## Introduction

Collaborative disaster management with various stakeholders has become imperative in disaster risk reduction because of future risks’ unpredictable and global nature. The socioecological approach advocates for disaster management to enhance community resilience, whereby communities engage in social learning from the encountered disasters, enabling transformative responses to future calamities (Berkes & Ross 2013; Imperiale & Vanclay 2021; Matarrita-Cascante et al. 2017; Ningrum, Chotib & Subroto 2022). This approach is highly suitable for countries that adhere to a dualism governance system, where the state governs the population, and collective decision-making is rooted in their ethnic or customary communities based on traditional norms of the nations (Holzinger, Kern & Kromrey 2016). At least 70 of the 2013 nations worldwide recognise the existence of specific ethnic, traditional or customary communities within their constitutions, with 61 countries explicitly acknowledging customary law and traditional governance systems in their governmental framework. Indonesia is among the nations that recognise the existing customary (known as *Adat*); *Adat* refers to local traditional systems of rights, belief and custom as they have evolved over time in different parts of Indonesia (Henley & Davidson 2007). In the current context, *Adat* practices are increasingly advocated for in disaster risk management, as evidenced by global agreements among nations under the Sendai Framework and developments in disaster studies. *Adat* norms, beliefs and behaviours are seen as potential contributors to preserving environmental and societal sustainability amid the trajectory of capitalism and emerging disaster risks, including managing the coronavirus disease 2019 (COVID-19) pandemic.

The challenges posed by significant disasters such as the COVID-19 pandemic include governmental readiness to face unforeseen, new and capacity-limited crises. This pandemic has engendered intricate and far-reaching risks, resulting in cascading effects that disrupt all of community life, affecting not only health but also social, economic and cultural dimensions ingrained within society. A viable solution to address these multifaceted predicaments lies in participatory management encompassing all aspects of existence. In disaster studies prompted by climate change, traditional communities wield considerable influence in effecting behavioural changes while interacting with ecosystems and transforming their traditional practices to contribute to disaster reduction initiatives (John 2015). Similar observations are evident during the COVID-19 outbreak, where, responding to shifts in social behaviour amid the pandemic, traditional leaders within customary communities play a significant role in mitigating the risks arising from the coronavirus, particularly in regions characterised by dualism in governance, such as Bali, Indonesia. The management of COVID-19 is intrinsically intertwined with the role of *Adat* government, as community engagement is driven by traditional leaders (in Balinese terms, it is the Prajuru) who facilitate behavioural changes during the pandemic (Ketut et al. 2022; Sari et al. 2021, 2022).

Despite the pervasive waves of modernisation and urbanisation throughout Bali, traditional-based leadership remains deeply ingrained in collective decision-making within the communities. Rooted in the teachings of Hinduism, which serve as a cohesive force within Balinese society, this connection continues to subject Bali’s populace to traditional governance, even though it is administratively integrated into the state’s governance structure. Regarding managing the COVID-19 disaster, the state’s inability to fully contain the spread of the virus has led to a decentralised approach to handling the pandemic, particularly within indigenous communities.

As emphasised in the Sendai Framework for Disaster Risk Reduction (SFDRR), collaboration between the state government and traditional institutions is crucial to optimise community risk reduction initiatives. Because of diverse vulnerabilities and capacities among communities, more than a centralised approach is needed to understand distinct risks and engage public participation effectively in reducing risks (Nkombi & Wentink 2022; Zamisa & Mutereko 2019). Critics challenge the efficacy of centralised disaster management, asserting that a command-and-control approach to disaster response fails to facilitate meaningful cognitive and interactive learning within communities during risk reduction endeavours (Imperiale & Vanclay 2019, 2021). A more intricate hurdle to fostering resilience within communities arises from governance dualism in certain regions, exemplified by Bali, where traditional leadership is pivotal in community mobilisation. This traditional leadership, extending beyond spiritual domains, encompasses intricate human–human and human–environment relationships. The investigation of disaster risk reduction within the paradigm of dualism governance, especially within Asian nations acknowledging traditional leadership, remains limited. This study aims to deeply analyse the COVID-19 disaster management system in a context characterised by dualism governance – a scenario wherein administrative control rests with the state. In contrast, traditional government and customary laws regulate diverse aspects of human life in Bali, Indonesia. We argue that addressing the COVID-19 pandemic should involve diverse stakeholders in harmonious collaboration, despite facing various challenges in regions where the population is governed by dualism in governance.

## Research methods and data

Utilising a qualitative approach, this study gathered primary data through in-depth interviews, focus group discussions (FGDs) and participant observation, employing a combination of purposive and snowball sampling procedures for informant selection (Neuman 2014). The purposive technique involved identifying informants engaged in COVID-19 disaster risk reduction efforts, including provincial government representatives acting as extensions of central governance, traditional or *Adat* authorities and impacted community members. The snowballing technique involved identifying potential informants based on predetermined criteria through referrals from previously interviewed informants (see Table 1). We conducted research from the early- to mid-2023 when the Balinese community was revitalising tourism efforts after a 3-year decline because of the COVID-19 pandemic.

**TABLE 1:** Techniques of data collection and distribution of interviews.

Techniques of data collection	Frequency	Participants	Duration
In-depth interviews	10	7 informants from state or administrative government 3 informants from traditional or *Adat* governance	1–2 h per informant
Focus group discussions (FGDs)	3	One FGD with communities Two FGDs with state or administrative government	2–3 h per FGD
Participant observations	3 (events)	Local business in Banjar Danginpeken Denpasar such as minimarket and food stall Management of the coastal area of Intaran Village, Denpasar Selling activities at Kuta Beach	1–2 days per event

The data analysis process involved transcribing all interviews and FGDs data. These transcripts were then organised into categories based on themes, concepts or specific similarities, a process known as coding (Neuman 2014). The researcher utilised NVivo 12 software to assist in the coding process. The authors also employed triangulation and member-checking techniques for data quality (Krefting 1991).

### Ethical considerations

Ethical clearance to conduct this study was obtained from the National Research and Innovation Agency Ethics Committee, Indonesia (No. 089/KE.01/SK/04/2023).

## Results and discussions

The dualism governance in Bali can be observed through two distinct forms of governance: the governance representing the state administration and the governance based on *Adat* territories (traditional villages or ‘*desa pakraman*’). The state administration or governmental structure emerges because of Bali’s status as one of the provinces within the Republic of Indonesia, wherein each province has a local government led by a governor and serves as an extension of the national or central government. On the other hand, traditional or *Adat* governance is a system that originated in the 9th century, guiding and directing affairs based on the territorial boundaries of traditional villages and possessing distinct customary regulations separate from the national rules (Suyadnya 2021).

According to Provincial Regulation of Bali, Number 4 of 2019 concerning *Adat* Villages in Bali, a *Adat* Village is a collective entity of customary law society in Bali, possessing its territory, original status, composition, traditional rights, independent wealth, traditions, code of social conduct and intergenerational societal interactions within the sacred precincts (in Balinese terms, it is *Kahyangan Tiga* or *Village Kahyangan*). It encompasses responsibilities, authorities, rights to self-governance and autonomy management. The *Adat* Village operates under a distinct governance structure separate from administrative villages. While administrative governance adheres to state-prescribed protocols, the *Adat* Village government (*the Prajuru*) prioritises and oversees customary practices within the Balinese community. Administrative governance is limited to population-related administration tasks such as issuing identification identity cards, social welfare and implementing government programmes. In contrast, traditional governance oversees the three pillars of life known as *Tri Hita Karana*. This concept of Balinese societal existence, influenced by Hindu teachings, encompasses the realms of divine connection (in Balinese terms, it is *Parahyangan*), human relations (*Pawongan*) and harmonious coexistence with nature (*Palemahan*). Here, dualism can be observed in two governance systems operating concurrently within a specific community occupying a particular territory.

In building community resilience to address the COVID-19 pandemic, the existing dualism will be analysed across multiple dimensions derived from research coding outcomes. These codings include responses to vulnerabilities within the community, the coexistence of traditional or *Adat* and state regulations, and the communication system established between administrative and *Adat* authorities in managing the COVID-19 situation.

## Response to community vulnerability during COVID-19

The risk of virus transmission during the pandemic is not solely dependent on the number of infected people; it also considers how vulnerable people and communities are in all facets of life, which can lead to losses and damages. Various studies have indicated that the susceptibility to COVID-19-related vulnerabilities is influenced by overt factors such as food scarcity because of income loss (Atmaja, Kusyati & Fukushi 2021; Mejia, Bhattacharya & Miraglia 2020), unsanitary living conditions, lack of water and hygiene facilities (Franco et al. 2020; Jamieson & Van Blerk 2021; Kimani et al. 2021) and inadequate healthcare resources (Caruso, Mela & Pede 2020; Cohen et al. 2020). In addition, indirect factors contributing to vulnerability during the pandemic encompass population density (Batabyal & McCollum 2023), erosion of governance trust (Maudrie et al. 2021; Wan 2021) and the COVID-19 stigma (Leach et al. 2020).

This study reveals that the most pronounced vulnerability experienced by the Bali community is the downfall of the tourism sector. Bali has undergone a considerable economic decline because of the COVID-19 pandemic. It is noted that the percentage of the population living in poverty in Bali increased during the pandemic, rising from 3.61% in September 2019 to 4.72% in September 2021 (BPS 2020, 2022). Furthermore, the poverty rate is higher in urban areas compared to rural regions. Historically, Bali heavily relied on the tourism sector as its primary driving force, encompassing transportation, accommodations, restaurants and other tourism-related services. Throughout the 2-year COVID-19 pandemic, the entire tourism sector shut down, triggering a domino effect on other sectors such as agriculture, craft production and trade. The community experienced loss of livelihood, a shortage of credit funding from *Adat* Villages, limitations on customary and religious activities because of lockdown and isolation policies, diminished financial support from *Adat* Villages, and constraints on hospitals, oxygen and healthcare personnel for COVID-19 cases. These findings are conveyed through the coding outcomes of the following informant:

‘During the onset of COVID, Kuta faced an extreme decline, even to the point of describing our community as suffering greatly. This was because many individuals were overly ambitious in venturing into the tourism sector and starting businesses. They initiated loan requests from our Village Credit Institutions [*LPD*], the largest in Bali, reaching up to 0.5 trillion [*Rupiah*]. However, our LPD experienced a direct impact due to the pandemic. It became difficult to afford meals, and we couldn’t even repay our loans to the LPD.’ (The Prajuru, Kuta Bali, 08 May 2023)

Bali’s tourism industry’s evolution is intrinsically linked to preserving its cultural traditions. Similarly, the continuity of customary practices is contingent upon the income derived from tourism activities. This phenomenon engenders a substantial reliance of the Balinese community on the *Adat*-based governmental system. The *Adat* becomes a religious and cultural pursuit and an economic catalyst driving the socioeconomic communities. Indeed, this study’s insights elucidate conflicts between the administrative government and traditional authorities concerning conservation areas and the initiation of tourism ventures within the Intaran Denpasar *Adat* Village.

The government designates conservation forest areas under state control. At the same time, traditional governance, adhering to traditional norms, regards specific locales as *Adat* territories available for utilisation by the community in line with their economic empowerment objectives. Findings from community-focused group discussions further reveal that in matters of collective affairs, encompassing economic activities and customary engagements that directly impact the community, the customary governance apparatus is perceived as more accessible to the populace than the central government. The social institutions forged within the *Adat* framework facilitate communication between the community and *Adat* authorities, fostering a more seamless dialogue than with the state. Traditional governance is regarded as possessing a deeper understanding of the vulnerabilities faced by the Balinese communities compared to the state, which is often perceived as overly bureaucratic. This phenomenon is understandable, given that the essence of *Adat* consistently underscores the principles of dialogue, consultation and negotiation among coequal constituents. In contrast, the state is inherently associated with hierarchical structures, authoritative directives, uniformity and centralised control. Consequently, the sense of solidarity and adherence prevalent within *Adat* societies surpasses the bonds between the state apparatus and its citizenry.

To mitigate vulnerabilities, the national government, through its social assistance programmes, provides aid following central standards, including food assistance for isolated residents, disinfection efforts and telemedicine. This assistance is centralised, and the lengthy bureaucratic processes often lead patients to lament the delayed arrival of aid. In some instances, the study found that financial support arrives even after individuals have been declared virus negative. In contrast, the community in Bali views the presence of traditional governance as being more responsive as a result of its close relationship with the local population. The role of *Adat* extends beyond cultural matters to encompass economic concerns. Notable examples include the emergence of community-driven economic empowerment initiatives, beach management practices and the assurance of territorial security for tourism-related activities. During the pandemic, traditional governance in Intaran Village even directly formulated COVID-19 prevention protocols, such as social distancing guidelines for religious ceremonies. At the onset of the pandemic, when the national administrative government had not yet issued clear policies for addressing COVID-19, customary governance stepped in.

Similarly, when the Balinese populace suffered income loss because of the tourism industry’s closure, *Adat* authorities ensured that cultural practices persisted within the community. These efforts aimed to maintain local economic activity. The financial reserves of the *Adat* Village were allocated to provide food assistance to individuals undergoing self-isolation before central government aid was available. The presence of traditional leaders plays a pivotal role in local governance, not solely within the cultural realm, but also in conflict resolution and addressing societal issues. These leaders facilitate access to the entire community for traditional governance and foster a communication framework to reduce COVID-19 risks, which extends between the state and *Adat* authorities.

## Customary traditional rules versus state in handling COVID-19

The COVID-19 pandemic is new, and the government’s response system is still maturing. In the initial stages of COVID-19 dissemination in 2020, the government issued policies gradually, encompassing physical distancing, partial lockdowns and social safety nets (Djalante et al. 2020; Roziqin, Mas’udi & Sihidi 2021). Regrettably, these policies were not universally embraced because of their top-down nature and a lack of cognitive awareness among the populace concerning the lethality of the COVID-19 virus. The establishment of COVID-19 task forces in each province across Indonesia was abruptly executed without a clear set of standard operating procedures (SOPs) or a comprehensive pandemic management framework. The emergence of the ‘indonesiaterserah’ joke in online media implies a disregard for health regulations and a propensity to downplay the seriousness of the COVID-19 pandemic (Roziqin et al. 2021). Concurrently, the evolving landscape witnessed the emergence of various COVID-19 variants, leading to a surge in positive cases. This surge strained the fragile healthcare system, exposing its limitations in handling large-scale and abrupt events. COVID-19 task forces established at the provincial level, including in Bali, grappled with the challenges of keeping pace with swiftly evolving policy dynamics.

The community’s exposure to significant disaster scale and vulnerabilities highlights the need for a top-down pandemic management approach. Collaborating with *Adat* authorities, the Bali government established the ‘Satgas Gotong Royong’ (Mutual Cooperation Task Force) following the Bali Provincial Government Regulation 05/SK/MDA-Prov.Bali/III/2020, requiring every *Adat* Village to establish this task force in conjunction with administrative and traditional village officials. The national government recognises leveraging the resources of traditional governance, notably the ‘Pecalang’, which oversees health protocol compliance and enforces lockdowns, as an effective measure to curb virus transmission. To bolster COVID-19 management at the *Adat* Village level, the government directed *Adat* authorities to formulate specific regulations within a framework called ‘*Gering Agung Pararem*’. These regulations cover pandemic-related religious and traditional practices and the governance of daily social conduct, with corresponding sanctions for violators.

For *Adat* authorities, the government’s invitation is accepted and acted upon, as stipulated by traditional regulations (‘*Awig-Awig*’ in Bali), which emphasise the necessity to respect and uphold the fundamental laws of the Republic of Indonesian. In instances of conflict, the authority of village regulations may appear weakened, as evidenced in the following interview transcript excerpt:

‘*Adat* Villages cannot exercise their full authority; they must comply with national law. So, we acknowledge national law because in our regulations, known as “*Awig-Awig*”, there is an article stating that we uphold the constitution. However, this is a concern for *Adat* Villages; sometimes, we recognise our own laws, but when they are brought into the national legal framework, it seems as though customary law becomes defeated, overshadowed.’ (The Prajuru, Denpasar Bali, 05 May 2023)

At the onset of the pandemic in March 2020, the customary authorities in Denpasar implemented their regulations, mandating the use of masks for all community members when venturing outside. Similarly, adjustments were made to traditional ceremonies, with the chief of traditional governance limiting the number of participants and modifying the ceremonial procedures and mediums to align with health protocols, thereby minimising the risk of transmission:

‘Before any official instructions, we had already formulated regulations by the end of March 2020, imposing fines on individuals not wearing masks. We established these measures well before the government issued similar regulations, and in fact, the government used our area as a model in Denpasar. The same approach was applied to religious ceremonies; we practised prayers while maintaining social distance. We altered how ceremonial water is administered, utilising designated tools rather than direct hand contact. Likewise, modifications were introduced to other ceremonial elements to mitigate transmission risks. Additionally, we closed down all 6 kilometres of beaches in our region to prevent gatherings.’ (The Prajuru, Denpasar Bali, 05 May 2023)

The experience of handling COVID-19 within *Adat* communities underscores the imperative of local initiatives in responding to emergent risks from disasters, given that state assistance often entails prolonged timelines and may not comprehensively address community-level issues. This experience in Bali parallels analyses found in African studies, where traditional leaders serve as official representatives of local communities, reducing citizens’ dependence on the state (Hagmann 2007). In disaster management, the role of traditional leaders has been acknowledged within the context of disaster risk reduction, particularly in post-disaster scenarios marked by substantial losses. However, this recognition coexists with the inherent likelihood of resource ownership disputes stemming from disparities between customary values and state interests in the governance of natural resources.

## The communication between government and traditional leader in COVID-19 risk reduction

Conventional disaster management assigns a significant role to the government in planning, leading emergency response, facilitating recovery and assuming overall responsibility. The increasing frequency, complexity and uncertainty of faced disasters have reshaped conventional disaster management approaches towards collaborative strategies, encompassing communication and collective decision-making (Kapucu & Garayev 2011; Lee 2020). Within the context of dualism governance in Bali, collaboration involves both the state administrative government and traditional governance. Historically influenced by customary systems, decision-making at the community level now necessitates intensive communication between *Adat* and administrative authorities to effectively mitigate pandemic risks. The dissemination patterns of the coronavirus, treatment modalities and isolation protocols fall under the disaster management expertise of the administrative government through its healthcare resources. In contrast, traditional governance possesses the capacity to mobilise its constituents and stimulate changes in social behaviour as the community faces the pandemic.

Establishing the ‘*Satgas Gotong Royong*’, a collaborative effort between administrative and *Adat* authorities, seeks to facilitate more seamless communication. The apparatus of customary governance, from the village head (*Bendesa*) and its hierarchy at the village level, can effectively communicate and mobilise the community through the established ‘*Banjar*’ system. Within Bali’s social structure, a ‘*Banjar*’ represents a group of individuals or a community living in a specific area bound by agreed-upon traditional rules. Each ‘*Banjar*’ is led by a leader (*Klian Banjar*) chosen through community deliberation. The aggregation of ‘*Banjars*’ constitutes a customary village, led by the *Bendesa*. The head of the *Adat* Village or *Bendesa* can mobilise the entire community through the leaders of the ‘*Banjars*’. Within Bali’s disaster management system, the preexisting communication framework within the customary system presents an opportunity for administrative authorities to collaborate in disaster management.

The ongoing communication between the administrative government and *Adat* authorities cannot be detached, as actors within the administrative government, particularly among indigenous Balinese individuals, are also integrated into the Balinese customary society within their residential areas. Culturally, they are bound by their identity and traditional regulations. This community attachment has long been relied upon for disaster management. The spirit of togetherness fostered by existing social institutions is often seen as a manifestation of the state’s successful disaster management. However, criticisms of the state’s disaster risk management stem from perceived budgetary priorities, which are considered insufficient. Disaster studies practitioners in Bali express concern that if the government does not give substantial priority to the disaster management system and continues to rely solely on the social capital already established within the customary community, future risk reduction efforts may become increasingly vulnerable amid potential disasters that may arise in Bali, whether because of pandemics, tsunamis, volcanic eruptions or even the frequent occurrence of terrorism in tourist areas. This sentiment is echoed in the statement of the informant:

‘It wouldn’t be fair to attribute effective disaster management in Bali solely to the government. Just recently, we held interdepartmental coordination meetings and evaluations. Interestingly, the government’s disaster management programs are limited. If addressed, this situation could pose significant risks to achieving resilience. Moreover, the preservation of social capital within these social institutions is subject to change, and hence, there is much to be done, particularly in strengthening infrastructure.’ (Disaster administrator, Denpasar Bali, 02 Mei 2023)

Future changes stemming from climate change, urbanisation, the erosion of traditional values among the younger generation, and conflicts over natural resource governance between the state and indigenous communities can engender vulnerabilities within communities, thereby exacerbating the risks associated with disasters. The involvement of indigenous communities throughout disaster management is imperative, not solely confined to the response phase during disasters but necessitating comprehensive communication and collaboration between the state and customary authorities across the planning, mitigation, preparedness, response and recovery phases. This holistic approach is essential for achieving resilience within the Balinese community.

## Conclusions

In this study, the authors describe and organise knowledge on the social phenomenon observed in managing the COVID-19 pandemic within a region characterised by dualism in governance. This dualism entails a governmental structure under the jurisdiction of the state alongside traditional governance based on customary laws. Existing literature on dualism governance primarily focuses on democratic systems, conflict, peace and the socioeconomic development of nations (Holzinger et al. 2016). However, it has to explore its implications for disaster management extensively. The contemporary conceptual framework promotes disaster management through a collaborative approach involving various stakeholders, driven by the escalating frequency, impact and uncertainty of disasters. Although commonly perceived as being concerned solely with cultural and religious matters, traditional governance is shown in this study to oversee all aspects of community life, spanning spiritual activities to social and economic realms.

Amid the emergence of COVID-19, where the entire government disaster system struggles to manage substantial losses, traditional governance plays a role not only in assisting the government to contain the spread of COVID-19 but also in independently seeking solutions to aid its citizens facing economic hardships because of the closure of tourism in Bali. While conflicts over natural resource management have arisen in traditional and state domains, a harmonisation emerges when responding to major disasters. This phenomenon is also evident in 19 other African countries, leading to the conclusion that dualism of governance power compels communities to adapt through the hybridisation of their political institutions, transcending the confines of the two political realms, particularly in times of significant disaster (Logan 2013). Conversely, the substantial social capital within traditional communities is sometimes attributed to the success of state-driven disaster management systems. The collaboration between the state and traditional communities should be strengthened during significant disasters and across all phases of disaster management, encompassing mitigation planning, preparedness, response and recovery. Additionally, this collaboration should foster robust community capacities to build resilience when facing unforeseen disasters.
